# Direct Self-Injurious Behavior (D-SIB) and Life Events among Vocational School and High School Students

**DOI:** 10.3390/ijerph15061068

**Published:** 2018-05-24

**Authors:** Lili O. Horváth, Maria Balint, Gyongyver Ferenczi-Dallos, Luca Farkas, Julia Gadoros, Dora Gyori, Agnes Kereszteny, Gergely Meszaros, Dora Szentivanyi, Szabina Velo, Marco Sarchiapone, Vladimir Carli, Camilla Wasserman, Christina W. Hoven, Danuta Wasserman, Judit Balazs

**Affiliations:** 1 Doctoral School of Psychology, Eotvos Lorand University, 1075 Budapest, Hungary; szentivanyi.dora@ppk.elte.hu (D.S.); szabina.velo@gmail.com (S.V.); 2 Institute of Psychology, Eotvos Lorand University, 1075 Budapest, Hungary; gyorido@gmail.com (D.G.); kereszteny.agnes@ppk.elte.hu (A.K.); balazs.judit@ppk.elte.hu (J.B.); 3 Pedagogical Services, Budapest District 12, 1126 Budapest, Hungary; maripen7@gmail.com; 4 Vadaskert Child and Adolescent Psychiatry Hospital and Outpatient Clinic, 1021 Budapest, Hungary; gyongyver.dallos@gmail.com (G.F.-D.); gadorosju@gmail.com (J.G.); meszaros.gergely.83@gmail.com (G.M.); 5 West Hertfordshire Specialist CAMHS St Albans Clinic, AL3 5TL St Albans, UK; lucafarkas@gmail.com; 6 Semmelweis University, Mental Health Sciences Doctoral School, 1083 Budapest, Hungary; 7 Department of Health Sciences, University of Molise, 86100 Molise, Italy; marco.sarchiapone@me.com (M.S.); vladimir.carli@me.com (V.C.); 8 Karolinska Institutet, SE-171 77 Stockholm, Sweden; danuta.wasserman@ki.se; 9 Global Psychiatric Epidemiology, Columbia University-New York State Psychiatric Institute, New York, NY 10032, USA; camillawasserman@gmail.com; 10 Department of Epidemiology, Mailman School of Public Health, Columbia University, New York, NY 10032, USA; christina.hoven@nyspi.columbia.edu

**Keywords:** direct self-injurious behavior, D-SIB, self-injury, self-harm, life events, adolescents, SEYLE, depression, anxiety disorders, suicidal behavior, suicide prevention

## Abstract

Although several studies have recently assessed direct self-injurious behavior (D-SIB) among adolescents, it is still understudied in adolescents attending vocational schools: an educational setting generally associated with lower socioeconomic status. After extending the “Saving and Empowering Young Lives in Europe” (SEYLE) project to a vocational school population, we examined their D-SIB and life event characteristics compared to the high school population. SEYLE’s Hungarian randomly selected high school sample (*N* = 995) was completed with a randomly selected vocational school sample (*N* = 140) in Budapest, Hungary. Participants aged 14–17 years completed the SEYLE project’s self-administered questionnaires. D-SIB lifetime prevalence was significantly higher (29.4%) in the vocational school group compared to the high school group (17.2%) (Χ^2^(1) = 12.231, *p*< 0.001). D-SIB was associated with suicidal ideation in the vocational school group. Different life events were more frequent in the high school than in the vocational school group, and associations between D-SIB and life events differed in the vocational school group compared to the high school group. In conclusion, vocational school students are a vulnerable population with a higher prevalence of D-SIB compared to high school students. Life events and their association with D-SIB also differ in vocational school students compared to high school students. Taking all these into account might contribute to prevention/intervention designed for this population.

## 1. Introduction

There is a growing scientific interest in self-injury [1,2], leading to increased knowledge in several areas, e.g., classification, prevalence, correlates, and functions of self-injury [3]. Based on such research, self-injury became an independent disorder worthy of further consideration in the 5th Edition of the Diagnostic and Statistical Manual of Mental Disorders (DSM-5) [4] in Section III. ‘Conditions for Further Study’.

According to the literature on self-injury, a great heterogeneity can be found regarding definitions and terms [2,5]. In this study, we follow Brunner et al. [6] and Koenig et al. [7] and use the term direct self-injurious behavior (D-SIB), defined as intentional self-inflicted damage to one’s body surface, regardless of suicidal intent.

Despite the heterogeneity in terminology, studies measuring D-SIB, regardless of suicidal intent, and those focusing on non-suicidal self-injury (NSSI) specifically, have found comparable prevalences among adolescents, suggesting that these studies probably measure similar phenomena [1].

Several risk factors have been described for D-SIB, including both internalizing and externalizing pathology [5], including depression, eating disorders, Axis II disorders, prior suicidal thoughts/behavior, exposure to peer D-SIB, and abuse [8].

Based on previous studies the age of onset for D-SIB is typically around 12–14 years [9], and its prevalence is consistently higher among adolescents than adults [2]. Community studies suggest approximately 4% lifetime prevalence among adults and 15–46% among adolescents [6,10,11], reaching as high as 40–80% in adolescent clinical samples [9,12,13]. Considering the low ratio of hospitalization/medical treatment of individuals engaging in D-SIB, it can be assumed that a significant amount of D-SIB behavior is hidden at the community level [14] especially among adolescents of low socioeconomic status (SES), who, despite increased need, have less access to healthcare [15].

Low SES is known to be a risk factor for adolescent mental health, in part, because it is associated with certain bio-psycho-social factors, including an increased amount of stressful life events and poorer access to education and healthcare, which potentially increases vulnerability to problems associated with poor mental health [16]. To date, however, there is limited literature assessing the relationship between D-SIB and SES. Low SES, for example, is associated with an increased number of stressful life events (e.g., parental divorce) that might act as risk factors, mediating the relationship between SES and certain psychological problems known to be comorbid with D-SIB, such as childhood depression [17]. Most published results suggest a reverse relationship between D-SIB and SES (e.g., [18,19,20,21]), and a number of studies have also found associations between stressful life events and D-SIB itself [22,23,24].

Previous research suggests that low SES in childhood may not only increase exposure to stressful events. As a distal risk factor, it might also increase vulnerability to stressors through pathways such as dysregulation of the immune and stress-response systems [25,26]. Moreover, stressful events might be associated with D-SIB not only as distal but also as proximal risk factors: based on the SEYLE Study, Kaess and colleagues [24] found that the number of life events in the past six months predicted the first onset of D-SIB in the following year, suggesting that interpersonal life events play a critical role in the development of D-SIB.

According to previous research, high school and vocational school students in Hungary have significant differences regarding their SES, sociocultural background and perspectives on their future life [27,28]. High schools provide the opportunity to take the national secondary school leaving examination, that is required as entrance criteria for all higher education institutes (e.g., universities), while vocational schools provide a vocational qualifying certificate. One-third of vocational school students are counted as underprivileged, compared to 13% of high school students [29]. According to the 2010 Health Behavior in School-aged Children (HBSC) study, SES of students’ families is related to the type of educational setting attended by their children [30]. Not independently from these differences, vocational school students also differ from high school students regarding their health behavior (e.g., increased risk for tobacco- alcohol and drug use [30,31]; unprotected sex [30]); report less favorable attitudes towards school and classmates and report more bullying [30]. However, so far, adolescents who attend vocational education, at least in Hungary, have remained absent in research regarding D-SIB.

In the “Saving and Empowering Young Lives in Europe” (SEYLE) study—which was funded by the European Union under the Seventh Framework Health Program—more than 12,000 European high school students were assessed from 179 randomly selected high schools within 11 countries: Austria, Estonia, France, Germany, Hungary, Ireland, Israel, Italy, Romania, Slovenia, and Spain. Sweden served as the coordinating center. One of the main aims of the SEYLE project was to collect baseline and follow-up data on health, well-being, and risk-behaviors, including D-SIB among European adolescents, thus compiling an epidemiological database [32].

By extending the SEYLE study, the primary aim of our study was to describe the vocational school population, compared to the high school population—in terms of D-SIB and life events (i.e., whether vocational school students experience more life events than high school students). Our secondary aim was to explore how D-SIB is associated with suicidal ideation in the vocational school group. Another aim of our study was to explore possible differences in the associations between life events and D-SIB. Our final aim was to screen vocational school students with acute suicidal risk (emergency cases) and to offer immediate help for those in need by referring them to specialized care services.

Our hypotheses were as follows:

**Hypothesis** **1.** *The lifetime prevalence of D-SIB is higher in the vocational school group than in the high school group.*

**Hypothesis** **2.** *The prevalence of life events six months prior to assessment is higher in the vocational school group than in the high school group.*

**Hypothesis** **3.** *D-SIB is associated with suicidal ideation in the vocational school group.*

## 2. Materials and Methods

In our data collection protocol, we followed the methodology of the SEYLE project [32,33]. The SEYLE study was a Randomized Control Trial (RCT), which was registered at the German Clinical Trials Register (DRKS00000214). Approval for this study was obtained from the Ethical Committee of the Ministry of Human Capacities in Hungary (protocol number: 24798/2013/EKU).

The full description of the methodology, including assessment, instruments, and intervention was previously published [6,32,33].

### 2.1. Participants and Data Collection

Inclusion criteria were students ages 13–18 attending secondary schools in Budapest, Hungary. Two school types: high school and vocational school were included. Schools from both school types were randomly selected. In this current study, the high school group includes the baseline Hungarian population of the SEYLE Study, which was collected from September 2009 to February 2010 [32]. Using the same survey instrument as used in the SEYLE Study, data from the vocational school group was collected in October and November 2013.

Parents and students received both oral and written information about the study protocol and were asked to give written informed consent. Students completed the self-report questionnaires (see below) within the confines of the classroom in the presence of research staff, providing the opportunity to ask questions if necessary.

For emergency cases (participants with acute suicide risk) we followed the methodology of the SEYLE project: students who were categorized as high risk for suicidal behavior according to cut-off criteria of the SEYLE project [32] were referred to specialized care services.

### 2.2. Measurements

Lifetime prevalence of D-SIB was measured with the modified version of the Deliberate Self Harm Inventory [34]. This 6-item version is based on the 9-item DSHI questionnaire by Bjärehed and Lundh [35], a shortened version of the 16 item DSHI that originated from the 17 item DSHI by Gratz [34]. The 6-item version comprises the same facets on frequency, severity, and duration of D-SIB; but D-SIB acts were combined and reduced to assess self-injurious behaviors to the body surface only [6,34]. The questionnaire measures lifetime prevalence for intentional self-cutting, -burning, -hitting, -scratching, -carving and -biting, as well as preventing wounds from healing and skin damage by other methods on a 4-point Likert scale (never, 1–2 times, 3–4 times, 5 or more times). Cronbach-alfa was 0.662 in the vocational school sample and 0.754 in the Hungarian high school sample.

Suicidal ideation (suicidal thoughts or plans) in the last two weeks were assessed with the 4-item Paykel Suicide Scale (PSS) [36]. Each item was rated from 0 (Never) to 5 (Always). The total score was obtained by summing the four items. A fifth question screened the frequency of suicide attempts during the past two weeks, where the pupils could give “yes” or “no” answers. If pupils responded “sometimes”, “often”, “very often” or “always” to the question: “During the past two weeks, have you reached the point where you seriously considered taking your life or perhaps made plans how you would go about doing it?”; and/or “Yes” to the question “Have you tried to take your own life during the past 2 weeks?” they were identified as emergency cases and immediately referred to a child psychiatrist for further evaluation.

The life events list was developed for the SEYLE study [24,32], based on former life events literature, mainly the Social Readjustment Rating Scale (SRRS) [37]; the Life Events Checklist (LEC) [38]; and the ALCES [39]. The list consists of 27 minor and major life events, from which participants indicate the ones they had experienced during the six months prior to assessment. Indication of other life events at the “other life event” option at the end of the list was also possible.

### 2.3. Statistical Analysis

All statistical analyses were performed using IBM SPSS Statistics 20.0 (IBM Corp. in Armonk, NY, USA). During the selection of statistical tests, it was considered that D-SIB values did not exhibit normal distribution [40].

Descriptive statistics are reported in the text. *T*-tests were applied for continuous variables and Chi-square test for categorical variables when examining group differences between the vocational school and high school groups.

All tests of hypotheses were considered statistically significant if the two-sided *p*-value was less than 0.05. Bonferroni correction was applied to control for multiple comparisons.

For D-SIB prevalence, a dichotomized variable was created based on the first five items of the D-SIB questionnaire (assessing D-SIB methods, but not medical treatment due to D-SIB) to determine D-SIB occurrence/absence. For assessment of gender differences in relation to D-SIB methods, D-SIB methods were classified as “cutting type”/“non-cutting type”. The cutting type consisted of behaviors of the first and third item of the D-SIB questionnaire (i.e., “ever intentionally cut wrist, arms, or other area(s) of body, or stuck sharp objects into skin such as needles, pins, staples” + “ever intentionally carved words, pictures, designs or other markings into skin, or scratched yourself to the extent that scarring, or bleeding occurred”), while other forms of D-SIB were classified as the non-cutting type.

Binary logistic regression was used to estimate the probability of D-SIB based on life events both in vocational school and in high school groups. Dependent variable was D-SIB occurrence. For this statistical testing, as a second step the 27 life events were categorized in 9 major categories, based on previous literature [22]: problems with or in the family; death of someone; trouble with police or law; problems with schoolwork; personal health problems; difficulties with romantic/sexual relationships; difficulties with friends and pregnancy.

## 3. Results

### 3.1. Sample

The vocational school sample consisted of 140 students. Forty percent were female, aged between 14 and 17, and the mean age was 15.21 ± 0.77 years (1 person did not indicate their age). In the high school group, 1009 students participated; 58.9% were female, aged 13–18 years, and the mean age was 15.01 ± 0.8 years.

For a matched sample, we have left out cases under and above the age range 14–17 from the high school sample (14 cases). This resulted in a sample of 995 participants in the high school group, with mean age 15.09 ± 0.75 years, 59.2% were female. Weighting for age and gender) were applied in this sample to match it with the vocational school sample.

To make sure that the weighting process was efficient, we conducted a comparability analysis. Before dropping the 14 cases under and above the age range 14–17, the two groups significantly differed both in age (*t* = −2.046; *df* = 1146; *p* < 0.041) and gender (*t* = −4.251; *df* = 1147; *p* < 0.001). After dropping the 14 cases, difference in age was not statistically significant anymore (*t* = 1.78; *df* = 1132; *p* = 0.075), and in gender we still observed a difference (*t* = 4.325; *df* = 1133; *p* < 0.001). After the weighting procedure, none of the variables differed between the two groups (*t* = 0.000; *df* = 1132; *p* = 1.0 for age; *t* = 0.065; *df* = 1133; *p* = 0.948 for gender).

In the high school population, 11 pupils (1.1%) were considered emergency cases, while 2 (1.4%) pupils in the vocational school population were considered emergency cases.

### 3.2. D-SIB in Vocational School Students

#### 3.2.1. Gender Differences among Vocational School Students

Examining all types of D-SIB among vocational school students, 25.64% of boys (*N* = 78) and 35.41% of girls (*N* = 48) engaged in some form of D-SIB during their lifetime. There were no significant differences in D-SIB lifetime prevalence between girls and boys (Χ^2^(1) = 1.37; *p* = 0.242).

The most common method of D-SIB was “cutting-type” methods, which we examined by merging two items of the D-SIB scale. 88.2% of girls and 60% of boys reported this form of D-SIB; this difference is still not significant (Χ^2^(1) = 3.71; *p* = 0.054). Distribution of D-SIB methods types by gender can be seen in Table 1.

#### 3.2.2. D-SIB and Suicidality in Vocational School Students

Among vocational school students, 123 participants completed both PSS and D-SIB questions, and 36 of them reported having ever engaged in D-SIB. Mean scores were M = 2.36; SD = 3.6 among those who had, and M = 0.44; SD = 1.08 among those who had not engaged in any form of D-SIB, which is a significant difference between the two groups (*t* = 4.54; *df* = 121; *p* < 0.01).

### 3.3. Differences between High School and Vocational School Students

#### 3.3.1. Lifetime Prevalence of D-SIB in Vocational and High School Students

**Figure 1:**
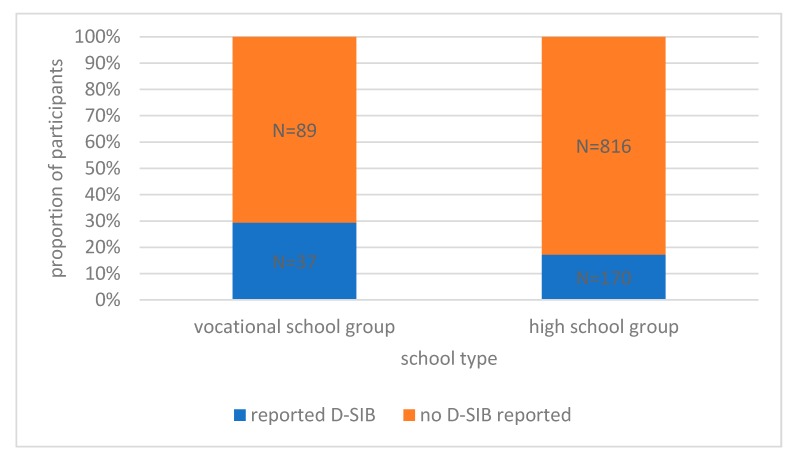
Lifetime prevalence of D-SIB in vocational school and high school groups.

In the high school group (*N* = 986) 17.2% of the adolescents and 29.4% in the vocational school group (*N* = 126) reported that they had engaged in some form of D-SIB (Figure 1). The two distributions differ significantly (Χ^2^(1) = 12.231, *p* < 0.001).

#### 3.3.2. Life Events in Vocational and High School Students

The following life events differed significantly in frequency of occurrence in the vocational school and high school groups: increased workload at school; appearing for an exam, interview; new family member; divorce between parents; death of a close friend and change of school.

Increased workload at school and appearing for an exam or interview were more frequent in the high school group. New family member; divorce between parents; death of a close friend and change of school were more frequent in the vocational school group.

Frequencies of occurrence of life events in the two groups and results of the Chi-tests are presented in Table 2.

#### 3.3.3. D-SIB and Life Events in Vocational and High School Students

As presented in Table 3, in the high school group (*N* = 995) when testing for associations between D-SIB and individual life events, D-SIB was significantly associated with trouble with parents and breakup with boyfriend/girlfriend (Cox and Snell R^2^ = 0.054; Nagelkerke R^2^ = 0.087; Omnibus test of model coefficients: Χ^2^ = 41.413, *df* = 26, *p* = 0.028; Hosmer and Lemeshow test: Χ^2^ = 8.443, *df* = 8, *p* = 0.391).

As presented in Table 4, among life events categories problems with or in the family; trouble with police or law and difficulties with romantic/sexual relationships were associated with D-SIB (Cox and Snell R^2^ = 0.058; Nagelkerke R^2^ = 0.094; Omnibus test of model coefficients: Χ^2^ = 45,575, *df* = 1; *p* < 0.001; Hosmer and Lemeshow test: Χ^2^ = 4,053, *df* = 7, *p* = 0.774).

As presented in Table 5, in the vocational school group (*N* = 140), no significant associations were found between either of the individual life events and D-SIB (Cox and Snell R^2^ = 0.155; Nagelkerke R^2^ = 0,226; Omnibus test of model coefficients: Χ^2^ = 19.339; *df* = 26, *p* < 0.822; Hosmer and Lemeshow test: Χ^2^ = 4.782, *df* = 7, *p* = 0.687) nor the life event categories and D-SIB (Cox and Snell R2 = 0.152; Nagelkerke R^2^ = 0.226; Omnibus test of model coefficients: Χ^2^ = 12,028; *df* = 10, *p* < 0.238; Hosmer and Lemeshow test: Χ^2^ = 7.624, *df* = 8, *p* = 0.471) (Table 6).

## 4. Discussion

To our knowledge, this is the first study to assess the characteristics—i.e., prevalence, gender differences, and associations with suicidal ideation—of D-SIB among vocational school students and to compare them with the characteristics of a sample of high school students. To our knowledge, our study is also the first to compare occurrence of life events in the two school types.

An alarming result of the current study is that almost one-third (29.4%) of the adolescents in the vocational school group reported that they had engaged in some form of D-SIB at some point in their life. This rate is not only significantly higher than the rate of adolescents in the Hungarian high school group who reported that they had engaged in some form of D-SIB at some point of their life, but it is also a remarkably high result compared to high school samples from European countries reported on the SEYLE Study. While the D-SIB prevalence rate of Hungarian high school students was the lowest reported among the participating SEYLE countries, the 29.4% reported by vocational school students in the current study can be considered high (SEYLE Study mean rate was 27.6%, with 17.1% being the lowest and 38.6% being the highest) [6].

Among vocational school students, 25.64% of boys and 35.41% of girls reported a lifetime history of some form of D-SIB. The difference of prevalence between boys and girls was not significant, either when assessing all methods, nor when assessing the most frequent cutting-type of D-SIB, which is usually associated with the female gender [41]. Thus, our results support no gender differences in the prevalence of D-SIB in the general population [3].

The current study identified a high prevalence of suicidal ideation among those students in the vocational school sample who had a reported history of D-SIB. These findings are consistent with previous studies which found associations with suicidality and D-SIB among high-risk youth [42], as well as results on possible functions (e.g., emotion-regulation, self-punishment, and anti-suicidal functions) of D-SIB [43].

Students in the two school types also differed in frequencies of certain life events. While adolescents in the high school group were more likely to report increased workload at school and appearing for an exam/interview in the six months prior to assessment, vocational school students were more likely to report having a new family member, change of school, divorce of parents and death of a close friend. Although some of these events occurred relatively infrequently in both groups, these results on the numbers and severity of life events support findings from studies that identified vocational school students as a group with increased vulnerability regarding several bio-psycho-social conditions [30,31].

According to our findings, it is not only the differences in quantity and quality of life events among vocational school students that contribute to differences in D-SIB prevalence between the two school types. Our results also show that association between life events and D-SIB differ in the two groups: in the high school group, D-SIB was associated with family problems with or in the family; trouble with police or law and difficulties with romantic/sexual relationships, while in the vocational school group, none of the life event categories were associated with D-SIB. Initially, this result might seem surprising, however taking the increased frequency of severe life events and the overall vulnerability to various bio-psycho-social risk factors in this group into account, these results suggest more complex patterns of risk factors in this population. Our results suggest that life events might be associated with D-SIB in various ways in groups with different socioeconomic backgrounds—without discarding the idea that personal experiences behind the same life event labels (e.g., new family member, change in health) can be very different in these populations. Targeted prevention and intervention might benefit from supporting adolescents and their environment (e.g., peers, potential gatekeepers) in coping with these issues. On a broader level, raising awareness among professionals (e.g., in-school and health care) about the differences in the challenging life events students might face can also contribute to increased effectiveness for prevention programs.

Our results need to be interpreted with the consideration of several limitations. The cross-sectional nature of our data does not provide information about causality, and thus longitudinal assessment of this population would be useful future research. Additional limitations are the use of only dichotomous variables that reduce the variability in the model, and using small sample size compared to the number of explanatory variables in the vocational school group. Furthermore, differences in SES between vocational and high school groups were not directly measured. However, we used randomly selected high schools and vocational schools, and as we have highlighted previously, former studies have shown that in Hungary, the SES of students’ families is related to the education type attended by their children [30]. A further potential limitation is that students who might be at the highest risk were more likely to be missed during recruitment of participants since consent also depended on adults (school staff and parents) with various levels of involvement and various attitudes towards mental health prevention. This lack of inclusion was also due to school absence and the dropout rates associated with the higher risk level in this population. Thus, real effect sizes might be deflated compared to the ones estimated in this study. Possible biases due to the methods of self-administered questionnaires in a classroom setting, despite research staff’s efforts to minimize these effects, should also be considered.

Despite these limitations, our study has several strengths. Vocational school students often stay hidden not only from the attention of researchers, but also from prevention and intervention programs. Thus, raising awareness of self-injury and related psychological and social problems in vocational schools, as well as screening and referring students who might need immediate help to specialized care services, was an important aim and actual outcome of our study. Importantly, our results can contribute to an increased effectiveness of targeted prevention and intervention programs for this population.

## 5. Conclusions

Vocational school students are a vulnerable group with high rates of D-SIB in both genders. These prevalences are higher than those reported by high school groups in the SEYLE study on an international level [6]. Vocational school students who engage in D-SIB also report increased rates of symptoms of suicidal ideation. Different life events are more frequent among high school than among vocational school students, and associations between D-SIB and life events might also differ in the two populations. These results indicate an urgent need for prevention and intervention programs for vocational school students. Taking school-type-specific patterns of psychosocial challenges into consideration when designing these programs might contribute to an increased effectiveness of targeted prevention and intervention.

## Figures and Tables

**Table 1:** Distribution of direct self-injurious behavior (D-SIB) methods types by gender.

	D-SIB Boys			D-SIB Girls			Total	
	Frequency (Number of Occasions)	*N*	%	Frequency (Number of Occasions)	*N*	%	*N*	%
Cutting or sticking sharp objects into skin	1–2	2	2.6	1–2	8	16.6	10	7.9
	3–4	1	1.3	3–4	2	4.2	3	2.4
	>5	1	1.3	>5	1	2.1	3	2.4
	total	4	5.1	total	12	25	16	12.7
Self-burning	1–2	3	3.8	1–2	0	0	3	2.4
	3–4	0	0	3–4	1	2.1	1	0.8
	>5	1	1.3	>5	0	0	1	0.8
	total	4	5.1	total	1	2.1	5	4.0
Carving or scratching skin	1–2	8	10.3	1–2	9	18	17	13.3
	3–4	3	3.8	3–4	1	2	4	3.1
	>5	1	1.3	>5	1	2	2	1.6
	total	12	15.4	total	11	22	23	18
Preventing wounds from healing/self-biting	1–2	4	5.1	1–2	4	8.3	8	6.4
	3–4	1	1.3	3–4	0	0	1	1.8
	>5	0	0	>5	0	0	0	0
	total	5	6.4	total	4	8.3	9	7.14
Head banging or self-punching	1–2	3	3.8	1–2	2	4.2	5	4.0
	3–4	0	0	3–4	0	0	0	0
	>5	0	0	>5	1	2.1	1	1.8
	total	3	3.8	total	3	6.3	6	4.8

**Table 2:** Life events among high school and vocational school students.

Life event in Past 6 Months	Occurrence in High School (*N* = 767) (%)	Occurrence in Vocational School (*N* = 128) (%)	Χ^2^	*df*	Sig.	ϕ
Increased workload at school	54.8%	33.9%	15.368	1	0.000 *	−0.132
Lower grades than expected	45.5%	39.1%	1.598	1	0.206	−0.043
Appearing for an exam, interview	36.6%	6.2%	46.745	1	0.000 *	−0.231
Trouble with parents	22.3%	17.2%	0.500	1	0.479	−0.02
Change in family member health	21.3%	14.1%	3.123	1	0.077	−0.060
Break up with girlfriend, boyfriend	16.3%	24.2%	6.914	1	0.009	0.089
Serious argument with close friend	16.3%	24.2%	7.911	1	0.005	0.95
Change in financial status of parents	13.7%	19.5%	3.793	1	0.051	0.066
Minor violation of law	11.3%	7.8%	2.798	1	0.094	−0.06
Parent stops or starts working	9.3%	12.5%	1.490	1	0.222	0.041
Death of close family member	6.3%	10.2%	4.820	1	0.028	0.074
Death of pet	6.0%	11.7%	5.616	1	0.018	0.080
Serious argument with a teacher	6.0%	7.9%	0.462	1	0.497	0.023
Theft of personal belongings	5.3%	6.2%	0.127	1	0.721	0.012
New family member	4.7%	11.7%	12.672	1	0.000 *	0.120
Alcohol or drug use by family member	3.4%	3.1%	0.065	1	0.799	−0.009
Major personal injury or illness	2.3%	3.1%	0.235	1	0.628	0.016
Trouble with bullies	2.2%	4.7%	1.090	1	0.297	0.035
Parents unemployed	1.4%	3.1%	2.237	1	0.135	0.050
Divorce between parents	1.3%	7.0%	16.767	1	0.000 *	0.138
Pregnancy	1.2%	0.8%	0.381	1	0.537	−0.021
Death of close friend	0.9%	4.7%	14.288	1	0.000 *	0.128
Marriage of emotionally close sibling	0.9%	2.3%	1.932	1	0.165	0.047
Failed important course, exam	0.8%	3.1%	3.436	1	0.064	0.063
Sex problems	0.8%	2.4%	4.646	1	0.031	0.073
Change of school	0.4%	7.9%	46.573	1	0.000 *	0.230
Jail term	0.1%	0%	2.042	1	0.153	0.048

* Bonferroni correction *p* < 0.05/27 = 0.003. (Χ^2^ = Chi square test value; *df* = degrees of freedom; Sig = significance; ϕ = Phi coefficient.

**Table 3:** D-SIB and individual life events in high school students.

	B	S.E.	Wald	*df*	Sig.	Exp(B)	95% CI for Exp(B)	
							Lower	Upper
Increased workload at school	0.087	0.208	0.174	1	0.677	1.091	0.725	1.641
Lower grades than expected	−0.041	0.207	0.039	1	0.843	0.960	0.639	1.441
Appearing for an exam, interview	0.255	0.203	1.576	1	0.209	1.291	0.867	1.922
Trouble with parents	0.498	0.240	4.309	1	0.038	1.645	1.028	2.631
Change in family member health	0.269	0.243	1.227	1	0.268	1.308	0.813	2.104
Break up with girlfriend. boyfriend	0.624	0.250	6.209	1	0.013	1.867	1.142	3.050
Serious argument with close friend	0.124	0.276	0.202	1	0.653	1.132	0.659	1.944
Change in financial status of parents	0.200	0.283	0.498	1	0.481	1.221	0.701	2.125
Minor violation of law	0.335	0.279	1.437	1	0.231	1.397	0.809	2.414
Parent stops or starts working	0.180	0.331	0.295	1	0.587	1.197	0.626	2.290
Death of close family member	−0.038	0.462	0.007	1	0.934	0.962	0.389	2.381
Death of pet	−0.035	0.430	0.007	1	0.935	0.966	0.416	2.245
Serious argument with a teacher	0.281	0.393	0.512	1	0.474	1.325	0.613	2.861
Theft of personal belongings	−0.330	0.460	0.514	1	0.474	0.719	0.292	1.772
New family member	−0.246	0.523	0.222	1	0.638	0.782	0.281	2.177
Alcohol or drug use by family member	−0.037	0.509	0.005	1	0.942	0.964	0.355	2.616
Major personal injury or illness	−0.433	0.715	0.367	1	0.545	0.649	0.160	2.633
Trouble with bullies	0.073	0.560	0.017	1	0.896	1.076	0.359	3.222
Parents unemployed	0.426	0.772	0.305	1	0.581	1.531	0.338	6.947
Divorce between parents	0.795	0.721	1.218	1	0.270	2.215	0.539	9.096
Pregnancy	0.974	0.771	1.594	1	0.207	2.647	0.584	11.999
Death of close friend	1.361	0.902	2.275	1	0.131	3.899	0.665	22.855
Marriage of emotionally close sibling	0.912	0.927	0.968	1	0.325	2.489	0.405	15.305
Failed important course, exam	1.985	1.492	1.772	1	0.183	7.282	0.391	135.489
Sex problems	−19.576	28,141.261	0.000	1	0.999	0.000	0.000	
Change of school	0.087	0.208	0.174	1	0.677	1.091	0.725	1.641
Constant	−2.044	0.197	107.764	1	0.000	0.130	−2.044	

B = unstandardized beta; S.E = standard error; Wald statistic. *df* = degrees of freedom; Sig = significance; Exp(B) = odds ratio; CI = Confidence Interval.

**Table 4:** D-SIB and life event categories in high school students.

	B	S.E.	Wald	*df*	Sig.	Exp(B)	95% CI for Exp(B)	
							Lower	Upper
Problems with or in the family	0.288	0.096	8.994	1	0.003 *	0.749	0.621	0.905
Death of someone	0.104	0.274	0.144	1	0.705	0.901	0.527	1.542
Trouble with police or law	0.616	0.274	5.054	1	0.025 *	0.540	0.316	0.924
Problems with schoolwork	0.044	0.103	0.179	1	0.672	0.957	0.782	1.172
Personal health problem	−0.337	0.681	0.246	1	0.620	1.401	0.369	5.320
Difficulties with romantic/sexual relationships	0.790	0.219	13.026	1	0.000 **	0.454	0.296	0.697
Pregnancy	1.386	0.762	3.308	1	0.069	0.250	0.056	1.113
Difficulties with friends	0.373	0.245	2.327	1	0.127	0.689	0.426	1.112
Bullied	−0.221	0.348	0.402	1	0.526	1.247	0.630	2.466

* *p* < 0.05; ** *p* < 0.001; (B = unstandardized beta; S.E = standard error; Wald statistic. *df* = degrees of freedom; Sig = significance; Exp(B) = odds ratio; CI = Confidence Interval).

**Table 5:** D-SIB and individual life events in vocational school students.

	B	S.E.	Wald	df	Sig.	Exp(B)	95% CI for Exp(B)	
							Lower	Upper
Increased workload at school	0.581	0.563	1.064	1	0.302	1.787	0.593	5.389
Lower grades than expected	0.244	0.529	0.213	1	0.644	1.276	0.453	3.599
Appearing for an exam, interview	−0.217	1.221	0.032	1	0.859	0.805	0.073	8.818
Trouble with parents	−0.437	1.032	0.179	1	0.672	0.646	0.085	4.883
Change in family member health	0.364	0.752	0.235	1	0.628	1.440	0.330	6.280
Break up with girlfriend. boyfriend	0.526	0.623	0.712	1	0.399	1.691	0.499	5.733
Serious argument with close friend	−0.971	0.783	1.539	1	0.215	0.379	0.082	1.756
Change in financial status of parents	0.997	0.682	2.136	1	0.144	2.711	0.712	10.329
Minor violation of law	−0.739	1.272	0.337	1	0.561	0.478	0.040	5.776
Parent stops or starts working	−0.360	0.887	0.165	1	0.685	0.698	0.123	3.967
Death of close family member	−0.502	0.885	0.321	1	0.571	0.606	0.107	3.432
Death of pet	−0.974	1.174	0.688	1	0.407	0.377	0.038	3.772
Serious argument with a teacher	0.372	1.305	0.081	1	0.776	1.450	0.112	18.709
Theft of personal belongings	−1.339	1.575	0.723	1	0.395	0.262	0.012	5.741
New family member	1.622	0.974	2.775	1	0.096	5.064	0.751	34.152
Alcohol or drug use by family member	1.838	1.662	1.224	1	0.269	6.286	0.242	163.283
Major personal injury or illness	−0.030	1.935	0.000	1	0.987	0.970	0.022	43.073
Trouble with bullies	−0.221	1.566	0.020	1	0.888	0.801	0.037	17.239
Parents unemployed	2.152	1.730	1.548	1	0.213	8.604	0.290	255.459
Divorce between parents	−0.479	1.361	0.124	1	0.725	0.620	0.043	8.918
Pregnancy	20.156	40,192.971	0.000	1	1.000	567,199,771.029	0.000	
Death of close friend	0.802	1.490	0.290	1	0.590	2.230	0.120	41.340
Marriage of emotionally close sibling	−1.179	2.290	0.265	1	0.607	0.308	0.003	27.377
Failed important course, exam	−0.015	2.426	0.000	1	0.995	0.985	0.008	114.325
Sex problems	0.391	1.171	0.111	1	0.739	1.478	0.149	14.682
Change of school	−1.494	0.403	13.767	1	0.000	0.225		
Constant	0.581	0.563	1.064	1	0.302	1.787	0.593	5.389

B = unstandardized beta; S.E = standard error; Wald statistic. *df* = degrees of freedom; Sig = significance; Exp(B) = odds ratio; CI = Confidence Interval.

**Table 6:** D-SIB and life event categories in vocational school students.

	B	S.E.	Wald	df	Sig.	Exp(B)	95% CI for Exp(B)	
							Lower	Upper
Problems with or in the family	0.319	0.326	0.957	1	0.328	0.727	0.384	1.377
Death of someone	−0.232	0.587	0.156	1	0.692	1.262	0.399	3.990
Trouble with police or law	−0.531	1.416	0.140	1	0.708	1.700	0.106	27.246
Problems with schoolwork	−0.221	0.270	0.669	1	0.413	1.248	0.734	2.120
Personal health problem	0.852	1.773	0.231	1	0.631	0.427	0.013	13.774
Difficulties with romantic/sexual relationships	0.764	0.682	1.256	1	0.262	0.466	0.122	1.772
Pregnancy	19.294	40,193.279	0.000	1	1.000	0.000	0.000	
Difficulties with friends	0.700	0.829	0.713	1	0.398	0.496	0.098	2.522
Bullied	0.175	1.041	0.028	1	0.867	0.839	0.109	6.460

B = unstandardized beta; S.E = standard error; Wald statistic. *df* = degrees of freedom; Sig = significance; Exp(B) = odds ratio; CI = Confidence Interval.
